# Comparación de la precisión de dos localizadores electrónicos apicales en piezas dentarias con perforaciones simuladas

**DOI:** 10.21142/2523-2754-0902-2021-055

**Published:** 2021-06-21

**Authors:** Teresa Flora Astocaza Reátegui, Lourdes Rocío Ayarza Flórez, Carmen Rosa García Rupaya

**Affiliations:** 1 División de Carielogía y Endodoncia, Carrera de Estomatología, Universidad Científica del Sur. Lima, Perú. tere.22694@gmail.com, rocioayarza@hotmail.com, gcarmen_rosa@hotmail.com Universidad Científica del Sur División de Carielogía y Endodoncia Carrera de Estomatología Universidad Científica del Sur Lima Peru tere.22694@gmail.com rocioayarza@hotmail.com gcarmen_rosa@hotmail.com

**Keywords:** endodoncia, dientes premolares, alginato, ápice del diente, endodontics, premolar teeth, alginates, tooth apex

## Abstract

**Objetivo:**

El propósito de este estudio fue comparar la precisión de los localizadores electrónicos apicales Propex Pixi y Raypex 6 en la determinación de la longitud de trabajo de piezas dentarias con perforaciones simuladas a diferentes niveles del conducto radicular.

**Materiales y:**

**Métodos:** Se utilizaron 36 premolares inferiores unirradiculares con un conducto, divididos aleatoriamente en tres grupos de 12 piezas dentarias cada uno. En el primer grupo, se realizaron dos perforaciones simuladas que fueron a nivel medio y a nivel apical; en el segundo grupo, se realizó una perforación simulada a nivel cervical, y en el tercer grupo, se realizó una perforación simulada a nivel apical. Se emplearon dos localizadores electrónicos apicales Propex Pixi y Raypex 6. La longitud real del conducto fue medida con un calibrador Vernier digital. Se aplicó estadística descriptiva y estadística inferencial con la prueba de Friedman a un nivel de confianza del 95%.

**Resultados:**

Al comparar la precisión de las longitudes obtenidas por los localizadores electrónicos apicales Propex Pixi y Raypex 6 en premolares inferiores con perforaciones simuladas a nivel medio y apical, premolares inferiores con perforación simulada a nivel cervical y premolares inferiores con perforación simulada a nivel apical, se encontraron diferencias estadísticamente significativas en la longitud real del conducto, p < 0,001, p = 0,008 y p = 0,006, respectivamente.

**Conclusiones:**

El localizador electrónico apical Propex Pixi (Dentsply Maillefer, Alemania) presentó mayor precisión en la determinación de la longitud real del conducto en piezas dentarias con perforaciones simuladas a diferentes niveles del conducto radicular.

## Abstract (en)

**Objective:**

The purpose of the study was to compare the precision of the Propex Pixi and Raypex 6 electronic apical locators in determining the working length of teeth with simulated perforations at different levels of the root canal.

**Materials and Methods:**

36 uniradicular lower premolars were used with a canal randomly divided into three groups of 12 teeth each. In the first group two simulated perforations were made at the middle and apical level. In the second group a simulated cervical perforation was made, and in the third group a simulated perforation was performed at the apical level. Two electronic apical locators Propex Pixi and Raypex 6 were used, and the real length of the canal was measured with a digital Vernier caliper. Descriptive and inferential statistics were applied with the Friedman test at a confidence level of 95%.

**Results:**

When comparing the precision of the lengths obtained by the Propex Pixi and Raypex 6 apical electronic locators, statistically significant differences were found in the real length of the canal (p= 0.006) between simulated perforation of lower premolars at the cervical (p<0.001) and the apical level (p=0.008),

**Conclusion:**

The Propex Pixi apical electronic locator (Dentsply Maillefer - Germany) presented greater precision in the determination of the real canal length in teeth with simulated perforations at different levels of the root canal.

## INTRODUCCIÓN

La intención del tratamiento endodóntico es eliminar los microorganismos de los conductos radiculares infectados mediante un procedimiento biomecánico combinado con terapia antibacteriana para lograr la cicatrización del tejido periapical. La unión cemento-dentina se considera al límite de seguridad para tener éxito en el tratamiento y conseguir el cierre biológico a expensas del cemento ^1^.

Las perforaciones radiculares son complicaciones que ocurren en un 3% a un 10% de los tratamientos endodónticos, según investigaciones de Zmener y Luberti ^2^. Estas perforaciones pueden estar en cualquiera de los tercios radiculares y frecuentemente son producidas por iatrogenias. El uso de localizadores electrónicos apicales (LEA) es importante para su diagnóstico y localización ^3^^,^^4^. Para determinar el nivel de las perforaciones radiculares, se observará si están en la raíz o en la corona del diente. De presentarse en la raíz, se localizan en el tercio cervical, tercio medio o tercio apical, y de presentarse en la corona, se localizan en el tercio cervical ^4^^,^^5^. Una perforación es una abertura artificial que crea comunicación entre el conducto radicular y los tejidos periodontales, lo que compromete el éxito del tratamiento endodóntico, además de ser uno de los accidentes más desagradables ^5^^,^^6^.

Los localizadores electrónicos apicales (LEA) son considerados las mejores herramientas para determinar la longitud real de los conductos radiculares durante el tratamiento endodóntico ^7^. Idealmente, los conductos radiculares se preparan y obturan hasta la constricción apical, que es la parte más estrecha con el diámetro más pequeño; es por eso que la precisión de un localizador electrónico apical en la longitud de trabajo resulta de vital importancia ^8^. Altunbas *et al*. ^9^ refieren que se propuso un método eléctrico para determinar la longitud del conducto; posteriormente, Sunada *et al.*^10^ introdujeron la corriente directa para medir la longitud del conducto, ya que la mucosa oral y el ligamento periodontal tienen la misma resistencia eléctrica para la determinación de la longitud de trabajo.

Con el paso de los años se han desarrollado varias generaciones de LEA. La primera se basó en la resistencia; la segunda, en la impedancia; la tercera utilizó múltiples frecuencias y la cuarta mide la resistencia y la capacitancia por separado, por lo cual utiliza una sola frecuencia a la vez ^3^^,^^11^^,^^12^.

Petrucci *et al.*^13^ afirmaron que las soluciones a base de electrolitos contienen iones libres que actúan como conductor eléctrico. Saraswathi *et al.*^14^ observaron que el rendimiento del alginato es superior para la evaluación de los LEA porque sostiene firmemente los dientes y permanece intacto durante la realización del estudio. Topuz *et al.*^15^ refirieron que el alginato tiende a deshidratarse rápidamente, por lo que sugiere realizar las mediciones dentro de los 30 minutos.

El propósito del presente estudio es comparar la precisión de los localizadores electrónicos apicales Propex Pixi y Raypex 6 en la determinación de la longitud de trabajo de piezas dentarias con perforaciones simuladas a diferentes niveles del conducto radicular.

## MATERIALES Y MÉTODOS

Este estudio fue aprobado por el Comité Institucional de Ética en Investigación de la Universidad Científica del Sur (CIEI-CIENTÍFICA) con el código N.º 245-2019-POS8. La muestra estuvo conformada por primeros premolares inferiores unirradiculares que fueron donados por el servicio de odontología del Centro de Salud Salas Guadalupe (Ica). 

El diseño fue de tipo experimental *in vitro*, mientras que, para determinar el tamaño de la muestra, se utilizó la fórmula de comparación de dos medias a un nivel de confianza del 95%, con poder estadístico al 90%, precisión del 2% y varianza obtenida en la prueba piloto de 1,58, lo que da como resultado 8 dientes por grupo. Para este estudio, la muestra constó de 36 premolares inferiores (PM) divididos en tres grupos de 12 dientes cada uno. El primer grupo tuvo PM con dos perforaciones una a nivel medio y otra a nivel apical; el segundo grupo, PM con una perforación a nivel cervical, y el tercer grupo, PM con una perforación a nivel apical. 

Fueron incluidos en el estudio premolares inferiores unirradiculares, de conductos rectos, con ápices cerrados, de un conducto y perforaciones simuladas a diferentes niveles del conducto radicular. Fueron excluidos premolares inferiores con ápices abiertos, reabsorciones, tratamiento endodóntico, hipercementosis, fracturados.

La investigadora (TF) fue capacitada por un especialista. Utilizó 8 dientes para la calibración interobservador y obtuvo un coeficiente de correlación intraclase (CCI) de 0,998 y 0,999 con los LEA Propex Pixi y Raypex 6; para la calibración intraobservador el CCI fue de 0,999 y 0,998 con los LEA Propex Pixi y Raypex 6.

**Figura 1:**
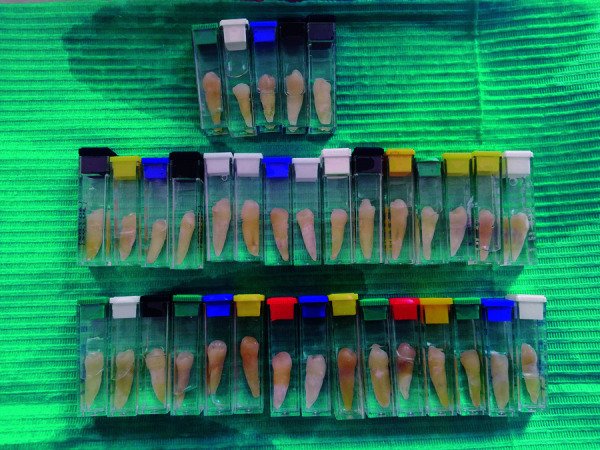
Premolares inferiores en frasco con suero fisiológico

Las piezas dentarias fueron desinfectadas por 2 horas con hipoclorito de sodio al 2,5%, y luego secadas y almacenadas en frascos con suero fisiológico (Figura 1). Además, se enumeraron en la pared vestibular.

**Figura 2:**
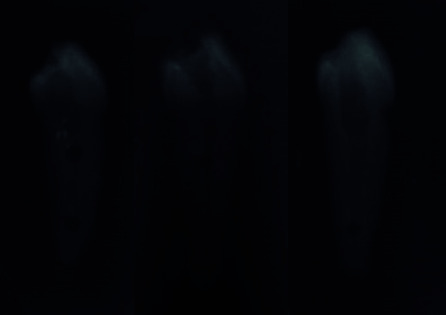
Radiografía periapical de las perforaciones simuladas a diferentes niveles

Primero, se realizó la apertura cameral; luego, la simulación de las perforaciones desde el exterior de la superficie de la raíz proximal hacia el espacio pulpar en un ángulo de 90º, con una fresa redonda ISO 012, y se comprobó las perforaciones con una radiografía periapical (Figura 2). La profundidad de la perforación a nivel cervical y nivel medio fue de 1,5 mm, y la perforación a nivel apical fue de 1 mm debido a la menor cantidad de tejido dentario en esta zona, según especificaciones de diversos autores ^11^^-^^13^^,^^16^.

**Figura 3:**
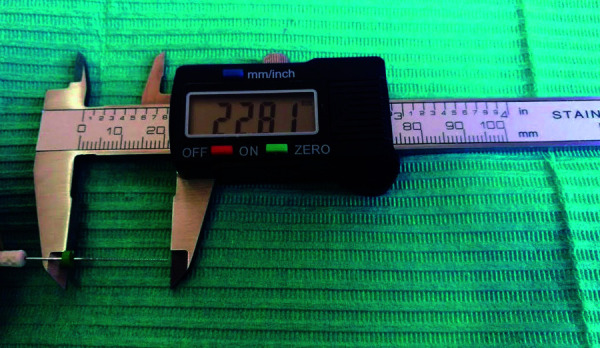
Medición de la lima n.^o^ 15 con el calibrador digital, desde el tope de goma hasta la punta de la lima

Se utilizó una lima n.º 15 para comprobar el trayecto del conducto radicular ^(3, 11)^. Posteriormente, la lima fue retirada y, con un calibrador milimétrico digital, se midió la distancia entre la punta de la lima y el tope de goma (Figura 3). A esta medida se le disminuyó 1 mm para obtener la longitud del conducto por visión directa, según lo mencionado por Echevarría y Saraswathi ^12^^,^^14^.

Se utilizó alginato para simular la cavidad oral ^10^^,^^11^^,^^17^ y este fue depositado en un recipiente de plástico donde se colocaron 6 dientes dejando 5 mm libres de la corona con un tiempo de trabajo de 30 minutos, según la recomendación de Topuz et al. ^15^. Se colocó el gancho labial y se humedeció el conducto con 0,2 ml de hipoclorito de sodio al 2,5% ^4^^,^^6^. Finalmente, se colocó el clip sobre la lima, a fin de cerrar el circuito y determinar la medición ^6^^,^^11^^,^^12^.

**Figura. 4:**
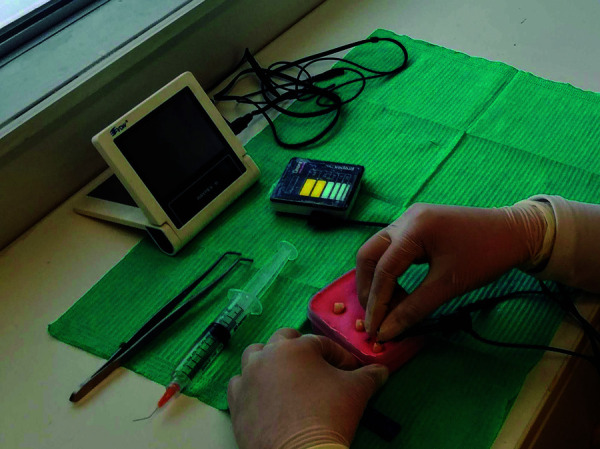
Medición del diente con el localizador electrónico apical Propex Pixi

**Figura 5:**
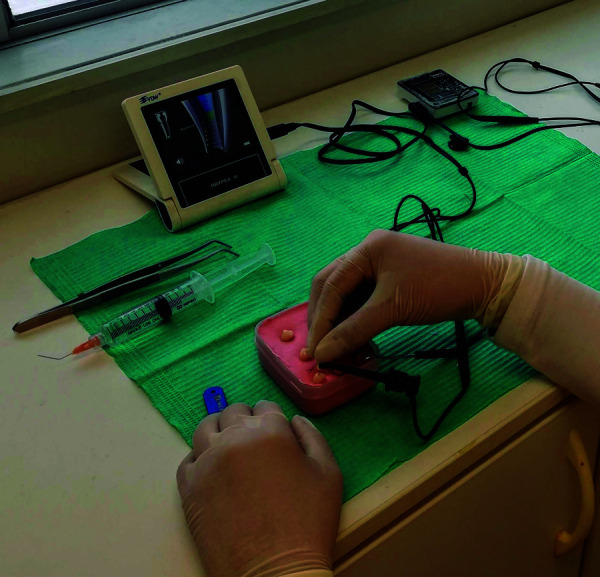
Medición del diente con el localizador electrónico apical Raypex 6

Según indicación del fabricante, al observar APEX (0,0) en el localizador electrónico apical Propex Pixi, se le restan 0,5 milímetros a la longitud obtenida (Figura 4). En el localizador electrónico apical Raypex 6, cuando se observan tres barras verdes marcadas en la pantalla esto significa que la lima ha llegado a la posición cercana al foramen apical (Figura 5) y se mide la longitud obtenida. Posteriormente, se registraron los valores obtenidos en una ficha recolección de datos para su evaluación.

Varios autores indican que las mediciones de los LEA se consideraron “precisas” cuando fueron de 0,50 mm; “aceptables”, entre 0,51 mm y 1 mm, y “equivocadas” si fueron mayores a 1 mm ^11^^,^^12^^,^^14^.

Mediante el uso del programa IBM SPSS Statistics 25, se realizó la estadística descriptiva que consistió en obtener la media, la desviación estándar y el rango de cada uno de los tres grupos. Posteriormente, para la estadística inferencial, se utilizaron la prueba no paramétrica U de Mann-Whitney y el análisis de varianza de Friedman para muestras relacionadas.

## RESULTADOS

Con respecto a la descripción de la longitud (mm) de las piezas dentarias obtenidas con los LEA y el tipo de perforación simulada en los diferentes niveles del conducto radicular, se obtuvo que el mayor valor encontrado de la media se halló en el grupo de LEA Raypex 6, con 21,74 mm, en dientes con una perforación a nivel cervical, y el menor valor se halló en el grupo de LEA Propex Pixi, con 20,16 mm, en dientes con dos perforaciones, una a nivel medio y la otra a nivel apical (Tabla 1).

**Tabla 1:** Descripción de la longitud (mm) de las piezas dentarias obtenidas con los localizadores Propex Pixi y Raypex 6 en perforaciones simuladas a diferentes niveles del conducto radicular

Localizador electrónico apical y nivel de perforación	n	Media	D. E.	Mínimo	Máximo
Propex Pixi perforación a nivel medio y nivel apical	12	20,16	1,26	18,42	23,16
Raypex 6 perforación a nivel medio y nivel apical	12	20,69	1,39	18,48	23,84
Propex Pixi perforación a nivel cervical	12	21,45	1,62	18,98	23,78
Raypex 6 perforación a nivel cervical	12	21,74	1,57	19,09	23,98
Propex Pixi perforación a nivel apical	12	21,21	1,47	19,38	23,75
Raypex 6 perforación a nivel apical	12	21,64	1,71	19,43	24,55

En la comparación de la longitud real del conducto con respecto a la precisión de la longitud (mm) de las piezas dentarias con dos perforaciones con los LEA Propex Pixi y Raypex 6, se encontraron diferencias estadísticamente significativas con un p < 0,05 (Tabla 2). 

**Tabla 2:** Comparación de las longitudes (mm) obtenidas con los localizadores Propex Pixi y Raypex 6 en perforaciones simuladas a diferentes niveles del conducto radicular con la longitud real del conducto

Localizador electrónico apical y tipo de perforación	Media	p
Longitud real del conducto	20,48	
Propex Pixi perforación nivel medio y apical	20,16	<0,001
Raypex 6 perforación nivel medio y apical	20,69	
Longitud real del conducto	21,52	
Propex Pixi perforación nivel cervical	21,45	0,001
Raypex 6 perforación nivel cervical	21,74	
Longitud real del conducto	21,43	
Propex Pixi perforación nivel apical	21,21	0,005
Raypex 6 perforación nivel apical	21,64	

Prueba Friedman

Con respecto a la precisión de las longitudes (mm) de las piezas dentarias en las diferentes perforaciones simuladas del conducto radicular, se observó que el localizador electrónico apical (LEA) Propex Pixi sobrestima la longitud en menos de medio milímetro en todos los casos y el LEA Raypex 6 subestima la longitud en menos de medio milímetro (Tabla 3).

**Tabla 3:** Descripción de la precisión de las longitudes (mm) de las piezas dentarias obtenidas con los localizadores Propex Pixi y Raypex 6 en perforaciones simuladas a diferentes niveles del conducto radicular

Localizador electrónico apical y nivel de perforación	n	Media	D. E.	Mínimo	Máximo
Precisión Propex Pixi perforación nivel medio y nivel apical	12	0,31	0,29	0,00	0,80
Precisión Raypex 6 perforación nivel medio y nivel apical	12	-0,21	0,32	-0,84	0,39
Precisión Propex Pixi perforación nivel cervical	12	0,08	0,14	-0,12	0,36
Precisión Raypex 6 perforación nivel cervical	12	-0,22	0,32	-0,84	0,19
Precisión Propex Pixi perforación nivel apical	12	0,22	0,28	0,00	0,80
Precisión Raypex 6 perforación nivel apical	12	-0,21	0,47	-0,98	0,76

En cuanto a la comparación de la precisión de los valores de la media de las longitudes (mm) de las piezas dentarias obtenidas con los LEA Propex Pixi y Raypex 6 en perforaciones simuladas a diferentes niveles del conducto radicular, se utilizó la prueba no paramétrica U de Mann-Whitney, mediante la cual se encontraron diferencias estadísticamente significativas con un p < 0,05 (Tabla 4).

**Tabla 4:** Comparación de la precisión de las longitudes (mm) de las piezas dentarias obtenidas con los localizadores Propex Pixi y Raypex 6 en perforaciones simuladas a diferentes niveles del conducto radicular

Precisión	Media	p
Propex Pixi perforación nivel medio y nivel apical	0,31	<0,001
Raypex 6 perforación nivel medio y nivel apical	-0,21	
Propex Pixi perforación nivel cervical	0,08	0,008
Raypex 6 perforación nivel cervical	-0,22	
Propex Pixi perforación nivel apical	0,22	0,006
Raypex 6 perforación nivel apical	-0,21	

Prueba U de Mann-Whitney

## DISCUSIÓN

El presente estudio empleó la radiografía periapical como herramienta de apoyo para corroborar la precisión de los LEA en la determinación de la longitud de trabajo. Respecto de la precisión de las longitudes de trabajo, el localizador Propex Pixi sobrestima la longitud y el Raypex 6 la subestima, en ambos casos en menos de medio milímetro. Cabe resaltar que la precisión se determinó por la diferencia encontrada entre la medida real del conducto radicular y la medida obtenida con el localizador electrónico apical (LEA).

El localizador electrónico apical Propex Pixi (Dentsply Maillefer) y el localizador electrónico apical Raypex 6 (VDW) presentaron diferencias significativas. Estos resultados difieren de los encontrados por Khatri *et al.*^3^, quienes demostraron que no existe diferencia significativa en perforaciones radiculares entre el localizador Gold VDW y el Localizador Raypex 6. Probablemente, la discrepancia de resultados se deba a que las perforaciones fueron realizadas en el lado distal de la raíz mesial de molares inferiores, porque es considerada zona de peligro de las piezas dentarias.

En el estudio realizado por Srivastava *et al*. ^5^ se menciona que el Raypex6 Apex locater detectó las perforaciones de la raíz de manera más significativa y puede ser mejor que Propex Pixi, a diferencia del presente estudio que muestra que el localizador electrónico apical Propex Pixi tuvo mejor precisión para determinar la longitud de trabajo en piezas dentarias perforadas artificialmente a diferentes niveles del conducto radicular. La similitud que presenta se consigue porque hay una coincidencia en el nivel de la perforación de ambos estudios.

Se obtuvieron valores negativos con medidas de precisión menores a medio milímetro utilizando el LEA Raypex 6 con una media de -0.21 mm, resultado parecido al del estudio de Gürel *et al.*^18^, quienes determinaron que Raypex 6 tuvo una media de 0,37 mm. La semejanza podría deberse al uso de la misma herramienta de medición y que, al ser de cuarta generación, logra una determinación eficaz de la longitud de trabajo.

El LEA Raypex 6 subestima la precisión de la longitud de trabajo en menos de medio milímetro y obtiene valores negativos dentro del rango de precisión -0,21 a -0,22 mm. El resultado encontrado por Khandewal ^19^ señala que la subestimación de la determinación de la longitud de trabajo con el LEA Raypex 5 fue de 0,72 mm. No se encontraron estudios que demuestren el porqué de la subestimación en la longitud de trabajo.

En este estudio se encontró que el localizador electrónico apical Propex Pixi (Dentsply Maillefer) sobreestima la precisión de la longitud de trabajo en menos de medio milímetro, con lo que obtiene valores positivos en la medición, semejantes a los del estudio de Somma *et al.*^20^, el cual menciona que los localizadores Raypex y ProPex dieron valores positivos y negativos utilizando una precisión de ± 0,5 mm de la longitud real del conducto. Los resultados obtenidos son similares porque utilizan LEA iguales para determinar la longitud de trabajo en dientes que presentan perforaciones.

Se recomienda realizar otros estudios con dientes perforados y el uso de irrigantes para conocer si influyen en la medición de la longitud de trabajo, así como otro tipo de LEA y comparar los resultados. También se pueden llevar a cabo estudios utilizando sangre de carnero, porque es similar a la del ser humano y permitirá determinar la efectividad de los LEA ^21^.

Estos resultados permitirán orientar al clínico a la elección más conveniente de un localizador electrónico apical en dientes que presenten perforaciones para obtener una determinación favorable de la longitud de trabajo. Cabe mencionar que, desde el punto de vista social, la selección de un buen localizador electrónico apical en estos casos puede ser muy beneficioso para los pacientes, ya que el tiempo de atención podría disminuir y serían atendidos eficazmente por el profesional.

## CONCLUSIÓN

En el estudio, se encontraron diferencias significativas entre el localizador electrónico apical Propex Pixi y el Raypex 6, que sobreestimaron y subestimaron, respectivamente, la longitud del conducto en menos de medio milímetro.

## Fuente de financiamiento:

Autofinanciado
